# Content Validity of Patient‐Reported Outcome Measures Developed for Assessing Disease‐Specific Quality of Life in Children With Sinonasal Disease: A Systematic Review

**DOI:** 10.1002/alr.23539

**Published:** 2025-02-19

**Authors:** Isabelle Williams, Oloruntobi Rotimi, Lisa Burrows, Annakan V. Navaratnam, Neil Tan

**Affiliations:** ^1^ Core Surgical Trainee, Guy's and St Thomas’ NHS Foundation Trust London UK; ^2^ University College Hospitals NHS Foundation Trust London UK; ^3^ Royal Cornwall Hospitals NHS Trust Truro UK; ^4^ University College Hospitals NHS Foundation Trust London UK; ^5^ University of Exeter Royal Cornwall Hospitals NHS Trust Truro UK

**Keywords:** health‐related quality of life, nasal cavity, paediatrics, paranasal sinuses, patient‐reported outcome measures, qualitative evaluations

## Abstract

**Objectives:**

Paediatric sinonasal disease is prevalent worldwide, giving rise to substantial healthcare costs and morbidity. However, there is a lack of sinus‐specific patient‐reported outcome measures (PROMs) tailored for children. This study aimed to evaluate existing PROMs against established validation criteria to determine whether a paediatric‐specific, validated instrument for assessing sinonasal disease currently exists.

**Methods:**

Medline (Ovid), Embase (Ovid), Emcare (Ovid), and the Cochrane Library were searched from inception to January 2024. Eligibility was restricted to PROM development or content validity studies conducted in all children with sinonasal disease. Content validity was assessed using the COnsensus‐based Standards for the selection of health Measurement INstruments (COSMIN) methodology, with evidence graded using a modified GRADE approach.

**Results:**

Five PROMs were identified: two for acute sinus disease (Sinus‐5 [S5] and Paediatric Rhinosinusitis Symptom Scale [PRSS]), two for general rhinosinusitis (Sinonasal‐5 [SN‐5] and Modified Sinonasal Outcome Test‐20 Young Persons Questionnaire [MSYPQ]) and one specific to primary ciliary dyskinesia (PCD; PCD quality of life [PCD‐QoL]). No content validity studies were included in the final analysis. The development of the S5, PRSS, SN‐5 and MSYPQ was found to be inadequate, supported by low‐quality evidence, whereas the PCD‐QoL stood out as a well‐developed tool created using established evidence‐based guidelines.

**Conclusions:**

Among the assessed tools, only the PCD‐QoL met standards for use in clinical trials, highlighting the need for a dedicated instrument to track disease status and QoL in otherwise healthy children with sinonasal disease.

## 1 Introduction

Sinonasal disease in children encompasses a wide range of conditions primarily inflammatory in nature, including viral rhinitis, acute rhinosinusitis, allergic rhinitis and chronic rhinosinusitis (CRS) [1].

**Summary**

- The quality of evidence for the content validity of paediatric‐specific patient‐reported outcome measures (PROMs) for evaluating sinonasal disease is currently insufficient.
- High‐quality evidence finds the primary ciliary dyskinesia (PCD)‐quality of life (PCD‐QoL) instrument as a well‐validated, rigorously developed tool for use in PCD patients.
- There is an urgent need to develop a disease‐specific PROM for sinonasal disease for children without PCD, adhering to COnsensus‐based Standards for the selection of health Measurement INstruments (COSMIN) methodologies.

CRS in children is prevalent, financially burdens families and healthcare systems due to its chronic nature [2], and can severely affect school performance and sleep quality [3]. In a large retrospective study by Cheng et al., children with CRS had greater than twofold increased odds of chronic school absenteeism, with even higher rates among those from low‐income households and with comorbid depression, anxiety and/or asthma [4].

Compared to traditional objective measures, patient‐reported outcome measures (PROMs) provide a more sensitive and relevant understanding of a patient's disease experience over time [5]. In children, a PROM serves as a cost‐effective tool for monitoring chronic disease status in real‐time, within a home environment, where decline in score(s) signals a change in disease state, initiating early or expedited review by a healthcare professional.

However, despite international initiatives to standardise PROMs in adult populations [6, 7], their use in paediatric settings is comparatively absent. Current PROMs utilised in clinical and/or research settings for evaluating paediatric sinonasal disease are adapted from adult measures or more broadly assess health‐related quality of life (HRQoL). The Sinonasal Outcome Test‐12 (SNOT‐12) instrument, derived from the SNOT‐22, has been validated as a screening tool for children with CRS, despite the original SNOT‐22 being developed only in adult populations [8]. Similarly, the SNOT‐16 has been used for symptom evaluation in a cystic fibrosis (CF) population aged 7‒20 years, despite no study validating its use in children [9]. General‐purpose questionnaires are more universally adopted to infer disease‐specific impacts of sinonasal and ENT disease, including the Paediatric Quality of Life Inventory 4.0 Generic Core Scales (PedsQL) [10, 11], the Child Health Questionnaire Parent Form (CHQ‐PF28) [12], the visual analogue score (VAS) [12, 13] and the EuroQoL 5‐Dimension Health Assessment (EQ5D) [14].

This systematic review uses the COnsensus‐based Standards for the selection of health Measurement INstruments (COSMIN) framework to assess if a paediatric‐specific PROM for sinonasal disease exists for use in clinical and/or research settings. This is a framework now well‐recognised for critically reviewing the quality and validity of PROM design [15].

## 2 Materials and Methods

This manuscript was written in accordance with the Preferred Reporting Items for Systematic Reviews and Meta‐Analysis (PRISMA) reporting guidelines [16].

### 2.1 Design

We performed a systematic review of content validity using the COSMIN methodology [15]. The protocol was registered in the PROSPERO database (CRD42024541040).

### 2.2 Search Strategy

The search strategy was developed by a Clinical Evidence Specialist (L.B.) together with a Consultant ENT surgeon (N.T.) and aimed to locate published and unpublished studies on existing PROMs validated for use in paediatric populations for sinonasal disease. The searches were run on January 9, 2024 in Medline (Ovid), Embase (Ovid), Emcare (Ovid) and the Cochrane Library. A further search of Cumulative Index to Nursing and Allied Health Literature (CINAHL) (EBSCO) did not identify any additional articles. Terms covered p(a)ediatric, sinonasal, PROMs and quality of life (QoL). Text terms and subject headings were combined with Boolean operators, with subject headings adjusted for each database. The search was limited to studies published in the English language as the authors did not have the resources for translations. No other limits were applied. The full search strategy is available in Appendix 1. Reference lists of included articles were examined to ensure that all relevant reports were captured.

### 2.3 Eligibility Criteria

Two blinded reviewers (I.W. and O.R.) independently screened studies using Rayyan. Eligibility was restricted to studies focused on the development or content validity of PROMs specific to sinonasal diseases in paediatric populations (aged naught to 18 years). Review articles were excluded. Included studies examined PROMs relating to acute, chronic and/or allergic (rhino)sinusitis, while those involving paediatric patients with CF or primary ciliary dyskinesia (PCD) were excluded.

A PROM development study was defined as any qualitative or quantitative research aimed at developing a PROM, including the pilot testing of draft instruments. A content validity study was defined as any investigation assessing the relevance, comprehensiveness or comprehensibility of an existing PROM based on input from patients and/or experts. These definitions align with the COSMIN framework.

Cross‐cultural adaptation studies were included only if the original PROM development study was determined to be of ‘adequate’ or ‘very good’ quality and included a pre‐test phase. A pre‐test was defined as a stage in PROM development whereby the instrument is tested on a small sample of the target population to evaluate reliability and validity before wider application. PROMs were included regardless of their intended context of use (evaluative, discriminative or predictive), defined as:

- Discriminative PROMs differentiate between individuals or groups at a specific time based on differences in health status, symptoms or QoL.
- Predictive PROMs aim to forecast future health outcomes or treatment responses based on baseline measurements.
- Evaluative PROMs track changes in health status over time, assessing disease progression, recovery or treatment effectiveness.

Due to the paucity of available literature, we adjusted our protocol to include PROMs validated in patients with CF or PCD.

### 2.4 Data Extraction

**FIGURE 1:**
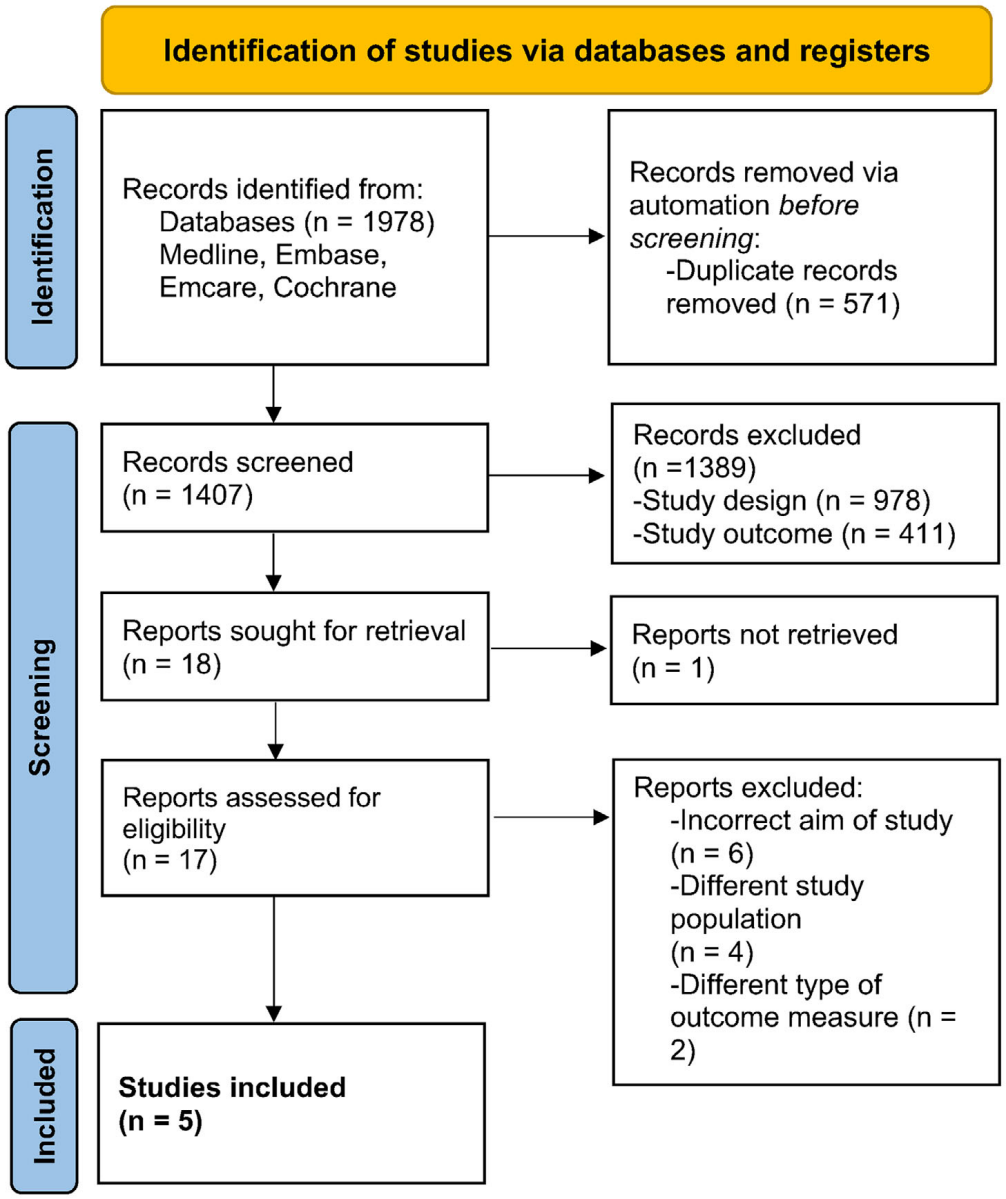
Preferred Reporting Items for Systematic Reviews and Meta‐Analysis (PRISMA) flow diagram for study selection. The figure outlines our process for identifying and selecting studies. Inclusion and exclusion criteria are clearly detailed.

Articles were initially reviewed based on title and abstract. Duplicates were removed and conflicts were discussed with a third, independent reviewer (N.T.). Articles considered relevant were reviewed in full text to establish a final set of studies to be included (Figure 1). Data were manually extracted including study design, number and sex of participants, disease, setting, country of origin, original language and patient/professional involvement (Table 1). PROM characteristics were isolated including construct of interest, origin of the construct, target population, context, (sub)scale(s), scoring and recall period (Table 2).

**TABLE 1:** Characteristics of included patient‐reported outcome measures (PROMs).

PROM	Construct(s)	Origin of construct(s)	Target population	Context of use	(Sub)scale(s), number of items ()	Scoring	Recall period (days)	Original language
S5	Acute sinus infection Nasal obstruction	SSQ, literature search, interviews with patients and professionals	Children with acute sinus disease (6 months–18 years)	Discriminative Evaluative	Blocked up or stuffy nose Headaches or face pain Coughing during the day Coughing at night	5‐point Likert scale	0.5, 3, 7, 10, 14	English
PRSS	Acute sinus infection Nasal obstruction	Questionnaires, interviews with patients and professionals	2–12‐year olds	Evaluative	Nasal symptoms (3) Cough (3) Malaise (2)	6‐point Likert scale	0 (symptoms over past 24 h)	English
SN‐5	Sinus infection Nasal obstruction Emotional functioning Activity limitation	OM‐6, literature search, interviews with children and caregivers	Children aged 2–12 years of age with stable sinonasal disease	Discriminative Evaluative	Sinus infection (7) Nasal obstruction (4) Allergy symptoms (4) Emotional distress (5) Activity limitation (3)	7‐point Likert Scale	0, 7, 4 weeks	English
MSYPQ	Sinus infection Nasal obstruction Sleep/fatigue Emotional functioning Physical functioning	SNOT‐20 (MSNOT‐20) questionnaire Suggestions from ethical committee	Children aged 11–16 years of age	Discriminative	Section 1: demographic data, ethnicity, family history (8 questions) Section 2: 20 disease‐specific items Section 3: quality of life/socioeconomic analysis (15 questions)	5‐point Likert scale	0	English
PCD‐QoL	Physical functioning Emotional and mental well‐being Social functioning Treatment burden HRQoL School/work performance	Literature search, open‐ended and cognitive interviews with patients/caregivers, professional input	Children (6–12 years), adolescents (13–17 years), caregiver proxy (child 6–12 years)	Evaluative	Child version (37 items, seven scales: physical, emotional and social functioning; treatment burden; upper and lower respiratory, ears and hearing symptoms) Caregiver proxy version (41 items, nine scales: same as child version, with additional health perception, eating and weight domains) Adolescent version (43 items, nine scales: as child version, with additional role and vitality domains)	5‐point Likert scale	0, 14 days	English

Abbreviations: HRQoL, health‐related quality of life; MSYPQ, Modified Sinonasal Outcome Test‐20 Young Person Questionnaire; OM‐6, Otitis Media‐6; PCD‐QoL, primary ciliary dyskinesia‐quality of life; PRSS, Parent‐Reported Paediatric Rhinosinusitis Symptom Scale; S5, Sinus‐5; SN‐5, Sinonasal‐5; SNOT‐20, Sinonasal Outcome Test‐20; SSQ, Sinus Symptom Questionnaire.

**TABLE 2:** Characteristics of included study populations in patient‐reported outcome measures (PROM) development studies.

PROM	Reference	Study design		Study size (*n*)	Mean age (SD, range (year)	Gender (% female)	Disease	Setting	Country	Language	Patient or proxy involvement?	Professional involvement?
S5	Garbutt et al. 1999 [17]	Cross‐sectional		1611	–	47	Sinus disease in *n* = 95	Single community paediatric ambulatory care practice	USA	English	No	No
		Prospective cohort		41	–	44	Acute sinus disease in *n* = 33					
PRSS	Shaikh et al. 2019 [18]	Prospective—study 1 (version 1.0)		258	6.4	48.1	All had clinically diagnosed acute sinus disease	Children presenting across six ambulatory paediatric clinics (single state)	USA	English	Yes—parents and children	Yes—experts on paediatric sinusitis
		Prospective—study 2 (version 2.0)		185	5.6	47.0		Children presenting across four different US states				
SN‐5	Kay and Rosenfeld 2003 [19]	Prospective		80	35.3 (6.7, 20.9‒49.3)	35	Rhinosinusitis	Single ENT practice	USA	English	No	No
MSYPQ	Sami and Scadding 2014 [20]	Cross‐sectional		20	–	–	CRS (EPOS 2007)	School	UK	English	No	Unclear
PCD‐QoL (child)	Dell et al. 2016 [21]	Prospective	Open‐ended interview	20	–	60	PCD (confirmed diagnosis)	Research centres, PCD clinics, support groups	UK, USA	English	Yes	Yes
			Cognitive interview	14		57.1						
PCD‐QoL (adolescent)			Open‐ended interview	20	–	40						
			Cognitive interview	16		43.8						
PCD‐QoL (parent‐proxy)			Open‐ended interview	29	–	–						
			Cognitive interview	17								

*Note*: ‘‒’ denotes absent or missing data.

Abbreviations: CRS, chronic rhinosinusitis; EPOS, European Position Paper on Rhinosinusitis and Nasal Polyps; MSYPQ, Modified Sinonasal Outcome Test‐20 Young Person Questionnaire; PCD‐QoL, primary ciliary dyskinesia‐quality of life; PRSS, Parent‐Reported Paediatric Rhinosinusitis Symptom Scale; S5, Sinus‐5; SD, standard deviation; SN‐5, Sinonasal‐5.

### 2.5 Evaluation of Content Validity and Risk of Bias Assessment

Content validity of PROMs was assessed in three stages. In step 1, the quality of the PROM development study was assessed using box 1 of the COSMIN framework using a four‐point scale: very good, adequate, doubtful and inadequate. In step 2, any available content validity studies were assessed using box 2 of the COSMIN framework. In step 3, the relevance, comprehensiveness and comprehensibility were evaluated based on the methods and results of PROM development, content validity studies if available, as well as the reviewers’ own ratings. This was done first per study (step 3a), before all available evidence on the relevance, comprehensiveness and comprehensibility was summarised and rated as sufficient (+), insufficient (−), inconsistent (±) or indeterminate (?) (step 3b). Finally, each rating of the content validity per PROM was accompanied by a grade for the quality of the evidence (high, moderate, low and very low), using a modified GRADE approach [22, 23], indicating how confident the reviewers were that the ratings were trustworthy (step 3c).

Two independent reviewers rated the studies in all three steps (I.W. and N.T.). The ratings were documented using a template Excel file available on the COSMIN website. The overall performance of items and scales were evaluated jointly, allowing for a better understanding of the overall effectiveness and relevance of the items in the context of the entire measure.

## 3 Results

Records were identified from four databases: Medline (Ovid), Emcare (Ovid), Embase (Ovid) and the Cochrane Library (Figure 1). A total of 1978 records were identified and uploaded to Rayyan. Five hundred and seventy‐one duplicate records were removed prior to screening. A total of 1407 records were subsequently screened and 1389 of these were excluded based on inclusion and exclusion criteria (Figure 1). A total of 17 papers were assessed for eligibility and the reviewers agreed on five articles for inclusion.

### 3.1 PROM Characteristics

Five PROMs were included in this review: the Sinus‐5 (S5) [17], the Sinus and Nasal Quality of Life (Sinonasal‐5 [SN‐5]) [19], the Paediatric Rhinosinusitis Symptom Scale (PRSS) [18], the Modified Sinonasal Outcome Test‐20 Young Persons Questionnaire (MSYPQ) [20] and the PCD‐quality of life (PCD‐QoL) [21]. Table 2 details the properties of each PROM.

The S5, derived from the Sinus Symptom Questionnaire, consists of five questions designed to assess acute sinonasal symptoms in children aged naught to 18 years. This instrument was designed specifically as a tool to recruit children with acute sinus disease into research studies.

The PRSS consists of eight items evaluating acute sinus disease symptoms in children aged 2‒12 years. Factor analysis classified symptoms into three domains: nasal, cough and malaise symptoms. It was designed primarily for assessing treatment‐related changes in clinical trials.

The SN‐5, based on the Otitis Media‐6 scale, was designed for use in 2‒12‐year olds with five domains evaluating disease‐specific symptoms, emotional function and physical activity. However, domain items are rated collectively, not individually. The instrument serves both discriminative and evaluative purposes.

The MSYPQ was adapted from the SNOT‐20 scale and targets 11‒18‐year olds. It includes three sections: demographic information, disease‐specific symptoms and general QoL. The disease‐specific section has 20 items divided into nasal, paranasal (ear and sinus) and sleep subgroups. The scale was primarily developed for discriminative use; however, it can also detect symptom change in response to treatment [24].

The PCD‐QoL, developed using rigorous conceptual frameworks, includes three tools for children (6‒12 years), their parent/carer proxies and adolescents (13‒17 years). Designed for clinical trials, it comprises seven subscales for the child version and nine for the adolescent and proxy versions. The final versions of the instruments with a breakdown of scale(s) and listed items were not detailed in the PROM development paper and were unable to be obtained from the authors [21].

#### 3.1.1 Step 1: Quality of PROM Development Studies

Table 1 details the PROM development studies involving a total of 2311 participants (children, adolescents and parent/carer proxies). Mean participant age varied and was inconsistently reported, with females comprising less than 50% of samples. All studies were conducted in populations representative of the target disease and all were initially developed in English. Demographic details for proxies were inadequately described, with ambiguity regarding whether gender and age referred to the proxy or the participant.

Table 3 summarises the overall quality ratings. The development of the S5, SN‐5 and MSYPQ was considered inadequate due to the lack of patient or proxy involvement. The PRSS design was rated adequate, but the absence of patient/proxy input on comprehensiveness together with poorly described interview methods led to an overall doubtful quality rating for its development. The S5, SN‐5 and MSYPQ were developed using children from a single institution, while the PRSS was based on a sample from six centres within a single US state. Consequently, these study populations were considered unrepresentative of broader paediatric populations.

**TABLE 3:** Quality of the patient‐reported outcome measure (PROM) development (ratings of box 1 of the COSMIN methodology) [15].

	S5	SN‐5	PRSS	MSYPQ	PCD‐QoL
Total quality of PROM design	I	I	A	I	V
Total quality of pilot study	I	I	D	I	V
Overall quality of PROM development study	I	I	D	I	V

Abbreviations: A, adequate; D, doubtful; I, inadequate; MSYPQ, Modified Sinonasal Outcome Test‐20 Young Person Questionnaire; PCD‐QoL, primary ciliary dyskinesia‐quality of life; PRSS, Parent‐Reported Paediatric Rhinosinusitis Symptom Scale; S5, Sinus‐5; SN‐5, Sinonasal‐5; V, very good.

Two independent reviewers assigned high‐quality ratings to the development of the PCD‐QoL using COSMIN criteria. The creation of three distinct questionnaires enabled the detection of nuances between younger children and adolescents. The PROM development process was rigorous, involving extensive literature searches, both open‐ended and cognitive interviews, and contributions from medical professionals, academics as well as international collaborators. A total of 116 individuals participated in the development process [25].

#### 3.1.2 Step 2: Quality of Content Validity Studies

Table 4 details content validity studies. Initially, six articles were included as content validity studies: four cross‐cultural adaptations of the SN‐5 (Turkish [26], Portuguese [27], Arabic [28] and Spanish [29]), an MSYPQ pilot study [24] and a psychometric evaluation of the PCD‐QoL [30]. However, all cross‐cultural adaptation studies for the SN‐5 were excluded due to inadequate PROM development. The MSYPQ pilot study lacked input from patients or professionals on the PROM's relevance, comprehensiveness and comprehensibility, leading to its exclusion. Similarly, due to lack of patient, proxy or professional involvement, the study from Behan et al. [30] evaluating the PCD‐QoL was excluded. No content validity studies were found for the S5 or PRSS.

**TABLE 4:** Characteristics of study populations in initially identified content validity studies, prior to exclusions.

PROM	Reference	Study design	Number of participants (*n*)	Age, mean (SD, range) (year)	Gender (% female)	Disease	Setting	Country	Language	Patient or proxy involvement?	Professional involvement?
SN‐5p	Uchoa et al. 2016 [27]	Prospective	51	5.82 (2.51, 2‒12)	45.1	CRS	Outpatient ENT clinic	Brazil	Portuguese	–	–
SN‐5a	Ragab et al. 2021 [28]	Prospective RCT	129	6.8 years ± 2.10	42.7	CRS (EPOS 2020)	Single ENT centre	Egypt	Arabic	No	Yes
Sp‐SN‐5	Calvo‐Henríquez et al. 2020 [29]	Prospective	154	7.86 (‒, 4.02–11.98)	58.4	Severe allergic rhinitis	ENT outpatient clinic, tertiary referral centre	Spain	Spanish	Yes (cognitive interview with 50 patients, pre‐test)	Yes
SN‐5t	Caytemel et al. 2022 [26]	Prospective	50	6.44 ± 2.73	38	CRS	ENT outpatient clinic	Turkey	Turkish	Yes (cognitive interview with 20 patients, pre‐test)	Yes (poorly described)
PCD‐QoL	Behan et al. 2019 [31]	Prospective validation study	Child (*n* = 71)	9.5 (2.04, ‒)	49.3	PCD	Survey (recruited from diagnostic centres/social media forums)	UK, North America	English	No	No
			Adolescent (*n* = 85)	15.4 (1.71, ‒)	48.2						
			Proxy (*n* = 68)	–	44.1						

*Note*: The lack of patient, proxy or professional involvement in the study by Behan et al., thus leading to its exclusion based on the COSMIN guidelines for inclusion of content validity studies.

Abbreviations: CRS, chronic rhinosinusitis; EPOS, European Position Paper on Rhinosinusitis and Nasal Polyps; PCD, primary ciliary dyskinesia; PCD‐QoL, primary ciliary dyskinesia‐quality of life; PROM, patient‐reported outcome measure; RCT, randomised control trial; SD, standard deviation; SN‐5, Sinonasal‐5.

Ultimately, no additional content validity studies aside from initial PROM development met the criteria for inclusion using COSMIN guidance.

#### 3.1.3 Step 3: Quality of the PROMs

Results from all relevant studies were qualitatively summarised to determine overall ratings for relevance, comprehensiveness and comprehensibility for each PROM, giving final ratings for content validity, accompanied by a quality of evidence grade.

The S5, PRSS, SN‐5 and MSYPQ were rated based on (very) low‐quality evidence, relying entirely on the reviewers' opinions under the COSMIN framework (Table 5).

**TABLE 5:** Analysis of content validity.

	Relevance		Comprehensiveness		Comprehensibility			
PROM	Ratings of results	Quality of evidence	Ratings of results	Quality of evidence	Ratings of results	Quality of evidence	Overall rating	Overall quality of evidence
S5	±	Very low	–	Very low	±	Very low	±	Very low
PRSS	±	Very low	–	Very low	+	Very low	±	Very low
SN‐5	±	Very low	–	Very low	±	Very low	±	Very low
MSYPQ	±	Very low	–	Very low	–	Very low	±	Very low
PCD‐QoL	+	Moderate	+	Moderate	+	Moderate	**+**	Moderate

Abbreviations: MSYPQ, Modified Sinonasal Outcome Test‐20 Young Person Questionnaire; PCD‐QoL, primary ciliary dyskinesia‐quality of life; PROM, patient‐reported outcome measure; PRSS, Parent‐Reported Paediatric Rhinosinusitis Symptom Scale; S5, Sinus‐5; SN‐5, Sinonasal‐5.

The PCD‐QoL received a sufficient (+) rating for relevance, comprehensiveness and comprehensibility, supported by both the PROM development study and reviewer ratings. While the absence of a content validity study prevented a very good evidence rating, the PCD‐QoL was felt to be rigorously developed with well‐described qualitative methods. Patients, proxies and professionals were involved from concept elicitation and the study benefited from a large, diverse sample [25].

Given that four of the five PROMs were deemed of inadequate quality, comparison and analysis of their psychometric properties was felt to be irrelevant and thus was not done.

## 4 Discussion

This study is the first to the authors’ knowledge to systematically review and document the availability, quality and use of PROMs specific for children with sinonasal disease.

Identifying the current state of paediatric PROM development in disease‐specific settings is essential for guiding future research efforts towards developing new instruments based on rigorous and standardised frameworks. The COSMIN methodology provides a systematic approach for evaluating the content validity of PROMs, with its detailed criteria enhancing the transparency and reproducibility of the evaluation process. Of note, quality ratings of PROM development can be shared via the online COSMIN database [15], meaning that the evaluative process need not be repeated multiple times in subsequent reviews.

### 4.1 Evaluation of Paediatric Sinonasal PROMs

This review identified five paediatric‐specific PROMs for sinonasal disease, with only the PCD‐QoL viewed to be of adequate quality, supported by good quality evidence. For all other PROMs, their development lacked involvement of patients, proxies and/or professionals, and thus their content validity remains at best, doubtful. As a result, the value of new interventions and treatments cannot be accurately determined or compared using these tools.

The SN‐5 is a widely used tool in paediatric CRS outcomes research, with existing data demonstrating improvements in scores following medical and surgical treatments [32]. However, the literature on QoL in paediatric CRS is limited, with very few studies providing intervention‐specific SN‐5 data in the general paediatric population [32]. While the SN‐5 demonstrates strong psychometric performance across four cross‐cultural adaptation studies (Table 4), the lack of patient or proxy and professional involvement rendered these findings obsolete in the evaluation of content validity. Like the S5, the SN‐5 was developed over a decade ago, prior to the adoption of advanced methodologies for concept elicitation and instrument development, raising questions about its future use in outcome‐based research.

Additionally, as the S5 was designed for discriminative purposes, it is unsuitable for monitoring changes in disease state over time or in response to interventions.

The development of the PCD‐QoL is robust, with appropriate scales for each paediatric cohort (children and adolescents), including proxies, developed using rigorous Food and Drug Administration‐endorsed qualitative methods and international collaboration [25].

Despite not meeting criteria for inclusion as a content validity study in our review (due to lack of patient, proxy and professional involvement), a large prospective study of 224 participants evaluated the validity and psychometric performance of the PCD‐QoL, with subsequent modifications to the scales [30]. Instrument development was meticulously detailed using multi‐trait analysis appropriate for the sample size. Psychometric testing confirmed the instrument as a robust, reliable and valid measure for use in children and adolescents [30]. The absence of floor effects and minimal ceiling effects implies good comprehensiveness, despite a lack of direct interview evidence [30]. Moreover, the authors allude to a series of teleconferences that led to the instruments being shortened by removing redundant items, supporting its relevance and comprehensibility [30]. Of note, while the PCD‐QoL provides a robust framework for assessing HRQoL in PCD patients, it is not sinus‐specific, lacking items relevant to sinonasal disease including nasal breathing, drainage or sinus pain.

### 4.2 PROM Design in Children

Developing a disease‐specific paediatric PROM presents unique challenges. Young children may require caregiver‐proxy reporting due to limited communication skills, potentially underestimating symptoms or impacts on QoL [33]. Additionally, chronic conditions in paediatric patients are dynamic, requiring PROMs to be sensitive to change over time. Measures adapted from adult PROMs (such as the S5 and MSYPQ) may miss important aspects of the paediatric experience, while ethical considerations often limit research in this area.

A paediatric‐specific PROM must capture all aspects of the paediatric experience at different developmental stages. The S5 scale, designed for children aged 6 months to 18 years, is unable to capture the nuances of developmental stages from infancy through to adolescence. Parent‐focused questionnaires are needed for young children and infants (naught to 6 years), while a mixed (combined) approach is more appropriate for children 6‒12 years. Patient‐centred questionnaires inclusive of QoL domains such as sleep, mood, schooling and relationships are needed for adolescent cohorts. The PCD‐QoL was the only scale that provided separate versions for children, young people and caregiver proxies.

### 4.3 Future PROM Development

We propose the development of a novel PROM designed to specifically evaluate sinonasal disease in children and adolescents, with a focus on capturing disease states longitudinally in a variety of clinical and research settings. Such a project must involve patients, caregivers and healthcare professionals to ensure the tool's relevance and accuracy, in line with COSMIN guidance.

As healthcare systems increasingly adopt remote modalities, with teleconsultation becoming standard practice, the use of paediatric PROMs for monitoring sinonasal conditions will be essential for identifying children who require referral and symptom evaluation. The integration of PROMs into digital platforms and mobile applications has the potential to empower children, adolescents and their caregivers to take more active roles in managing their health, while also enabling healthcare professionals to monitor patients more efficiently, both in person and remotely. Moreover, such a tool would serve as a standardised outcome measure for use in clinical trials evaluating new treatments and interventions, reflecting a true impact on the patient experience. Indeed, the PCD‐QoL is currently being utilised as an outcome measure in a clinical trial of azithromycin prophylaxis [34] as well as in a trial of VX‐371 versus ivacaftor [35].

### 4.4 Limitations

#### 4.4.1 Heterogeneity

The authors expanded their search to include all sinonasal disease patients, including those with systemic congenital conditions (CF and PCD). Heterogeneous reporting and methodologies made it difficult to compare study populations and PROM development methods. For example, the age range of the target populations varied significantly. Given that disease burden and symptomatology are likely to differ across paediatric age groups as well as between those with acquired disease versus congenital/genetic, such variability complicates meaningful comparisons between the PROMs included in this review.

#### 4.4.2 Missing Data

The authors were unable to retrieve the complete versions, including detailed breakdowns of the (sub)scale(s), of the MSYPQ and PCD‐QoL instruments. Therefore, these scales were evaluated in their entirety rather than by individual (sub)scale(s). This limitation is unlikely to significantly affect the overall conclusions of the study as neither meta‐analysis was performed nor was there an evaluation or comparison of the psychometric performance of PROM scales or items.

#### 4.4.3 Use of COSMIN Assessment Framework

The S5, SN‐5 and PCD‐QoL were all developed prior to the COSMIN initiative [15]. The COSMIN framework relies heavily on detailed documentation of methodologies and suboptimal reporting thus made the assessment of content validity challenging. Additionally, the framework is limited by the subjectivity of its evaluations which rely on qualitative judgments rather than objective, quantifiable metrics. However, this subjectivity reflects the inherent nature of health outcomes and perceived QoL, which are deeply personal and not always measurable through objective means. Improvements meaningful to patients may not be quantifiable, highlighting the importance of PROMs in being able to capture patients’ lived experiences and priorities.

## 5 Conclusions and Next Steps

At the time of writing this review, the PCD‐QoL is the *only* well‐developed and validated PROM for use in paediatric patients with PCD. Its development, adhering to COSMIN standards for content validity, offers a valuable model for the development of a new disease‐specific instrument for paediatric sinonasal disease outside of the PCD population.

## Conflicts of Interest

The authors declare they have no conflicts of interest.

## Supporting information



Supporting Information
