# Supplementary material for: Content Validity of Patient‐Reported Outcome Measures Developed for Assessing Disease‐Specific Quality of Life in Children With Sinonasal Disease: A Systematic Review
Source: Int Forum Allergy Rhinol. 2025 Feb 19;15(3):317–27. doi: 10.1002/alr.23539 (PMC11872780; doi:10.1002/alr.23539)
Supplement: Supplementary file 1 — Supporting Information [file ALR-15-317-s001.docx]

**Appendix 1: Search strategy re-run in Embase from inception to September 2024.**

Embase <1974 to 2024 September 27>

1            "infan*".ab,kf,ti.             624759

2            "toddler*".ab,kf,ti.         20644

3            "child*".ab,kf,ti.             2236896

4            "teen*".ab,kf,ti.              52729

5            "adolescen*".ab,kf,ti.  510298

6            "p?ediatric*".ab,kf,ti.   804446

7            "baby*".ab,kf,ti.             73279

8            babies.ab,kf,ti. 63708

9            "neonat*".ab,kf,ti.         440470

10          "boy*".ab,kf,ti. 248695

11          "girl*".ab,kf,ti.  243989

12          "young* person*".ab,kf,ti.         8143

13          "young* people".ab,kf,ti.           60510

14          exp child/          3253982

15          exp adolescent/            1875624

16          exp pediatrics/ 132211

17          1 or 2 or 3 or 4 or 5 or 6 or 7 or 8 or 9 or 10 or 11 or 12 or 13 or 14 or 15 or 16         5199376

18          "sinus and nasal quality of life survey".ab,kf,ti.              24

19          (SN-5 or SN-5H).ab,kf,ti.            120

20          "Sino-nasal outcome test".ab,kf,ti.       1284

21          SNOT-22.ab,kf,ti.          1809

22          "Rhinosinusitis Disability Index".ab,kf,ti.           125

23          RSDI.ab,kf,ti.    113

24          "mini-Rhinoconjunctivitis Quality of Life Questionnaire".ab,kf,ti.              95

25          MiniRQLQ.ab,kf,ti.         73

26          (NOSE adj1 (scale or score or questionnaire)).ab,kf,ti.              555

27          "Nasal Obstruction Symptom Evaluation".ab,kf,ti.       555

28          "Patient Reported Outcome Measure*".ab,kf,ti.            21346

29          PROMs.ab,kf,ti.              9388

30          "quality of life".ab,kf,ti.              664200

31          QOL.ab,kf,ti.     107991

32          outcome assessment*.ab,kf,ti.             17500

33          exp patient-reported outcome/             67555

34          exp "quality of life"/      719720

35          exp outcome assessment/       932656

36          18 or 19 or 20 or 21 or 22 or 23 or 24 or 25 or 26 or 27 or 28 or 29 or 30 or 31 or 32 or 33 or 34 or 35          1765338

37          sinusitis.ab,kf,ti.            25989

38          rhinosinusitis.ab,kf,ti.  17709

39          exp paranasal sinus disease/  66128

40          (inflamm* adj1 (nose or nasal or paranasal or sinus or mucosa)).ab,kf,ti.          2169

41          (paranasal sinus adj (disease or disorder)).ab,kf,ti.      347

42          37 or 38 or 39 or 40 or 41           73718

43          17 and 36 and 42           1615
